# Glutathione and Selenium Supplementation Attenuates Liver Injury in Diethylnitrosamine-Induced Hepatocarcinogenic Mice by Enhancing Glutathione-Related Antioxidant Capacities

**DOI:** 10.3390/ijms252111339

**Published:** 2024-10-22

**Authors:** Yung-Fang Hsiao, Shih-Chien Huang, Shao-Bin Cheng, Cheng-Chin Hsu, Yi-Chia Huang

**Affiliations:** 1 Department of Nutrition, Chung Shan Medical University, Taichung 40201, Taiwan; summerbreathsrelax@gmail.com (Y.-F.H.); chienchien2011@gmail.com (S.-C.H.); king@csmu.edu.tw (C.-C.H.); 2 Department of Nutrition, Chung Shan Medical University Hospital, Taichung 40201, Taiwan; 3 Organ Transplantation Center, Taichung Tzu Chi Hospital, Buddhist Tzu Chi Medical Foundation, Taichung 42743, Taiwan; sbc082366@gmail.com; 4 School of Medicine, Chung Shan Medical University, Taichung 40201, Taiwan

**Keywords:** glutathione, selenium, glutathione peroxidase, oxidative stress, hepatocarcinogenesis

## Abstract

Excess oxidative stress and inadequate antioxidant capacities are critical features in the development of hepatocellular carcinoma. This study aimed to determine whether supplementation with glutathione (GSH) and/or selenium (Se), as antioxidants, attenuates diethylnitrosamine (DEN)-induced hepatocarcinogenesis in mice. C57BL/6J male mice were randomly assigned to control, DEN, DEN + GSH, DEN + Se, and DEN + GSH + Se groups for 20 weeks. Daily supplementation with GSH and/or Se commenced in the first experimental week and continued throughout the study. DEN was administered in weeks 2–9 and 16–19 of the experimental period. DEN administration induced significant pathological alterations of hepatic foci, evidenced by elevated levels of liver function, accompanied by high malondialdehyde (MDA) levels; low GSH levels; and glutathione peroxidase (GPx), glutathione reductase (GR), and glutathione *S*-transferase (GST) activities. Supplementation with GSH and Se significantly ameliorated liver pathological changes, reducing liver function and MDA levels while increasing GSH levels and GPx, GR, and GST activities. Notably, combined supplementation with GSH and Se more effectively increased the GSH/glutathione disulfide ratio and GPx activity than individual supplementation. Supplementation with GSH and Se attenuated liver injury in DEN-induced hepatocarcinogenic mice by enhancing GSH and its related antioxidant capacities, thereby mitigating oxidative damage.

## 1. Introduction

Among all cancer types, liver cancer ranks as the sixth most common malignancy by incidence, with more than 86,000 new diagnoses reported each year, particularly prevalent in eastern Asia [1,2,3]. Hepatocellular carcinoma (HCC) accounts for more than 80% of primary liver cancer cases and is a leading cause of cancer-related death [2]. Excess reactive oxygen species (ROS) potentially overwhelm endogenous antioxidant defenses, leading to cell damage and genomic instability, which ultimately contribute to the development of HCC [4]. Therefore, maintaining the balance between oxidative stress and antioxidant capacities is a potential strategy for preventing the development of HCC.

Glutathione (GSH), a tripeptide comprising glutamine, cysteine, and glycine, is an important endogenous antioxidant that is primarily synthesized and utilized in the liver. Cysteine is a major limiting substrate in GSH synthesis. Because of the thiol group (–SH) provided by cysteine, GSH potentially captures and reduces ROS to less harmful metabolites. Moreover, it may be oxidized to glutathione disulfide (GSSG) via catalyzation by glutathione peroxidase (GPx). GSSG is subsequently reduced to GSH with the aid of glutathione reductase (GR) [5]. Numerous antioxidant supplements, such as thymoquinone and fucoxanthin, have been proven to exert a protective effect against diethylnitrosamine (DEN)-induced hepatocarcinogenesis in rats by retaining GSH and its related antioxidant capacities, including those of GPx, GR, and glutathione *S*-transferase (GST), while alleviating oxidative stress [6,7]. Supplementation with N-acetylcysteine (NAC), the precursor of GSH, early after DEN administration has proven to mitigate DEN-induced HCC tumor growth in mice through maintaining adequate levels of GSH and GST, without affecting the expression of nuclear factor-erythroid 2-related factor 2 (Nrf2), a key up-stream transcription factor of GSH metabolism and antioxidant capacities [8]. In contrast, recent research has indicated that GSH or NAC supplementation may directly reduce ROS levels and desensitized Nrf2/glutamate-cysteine ligase catalytic subunit (GCLC)-related antioxidant production pathway to promote HCC formation and tumor growth in vitro and in vivo [9]. Our previous study, and those of others, also revealed higher GSH levels and GPx activities [10,11] and lower lipid and DNA peroxidation product levels [12] in human HCC tissue than in adjacent normal tissue specimens. The role GSH plays as a suppressor or promoter during HCC development warrants further elucidation.

Among GSH-related antioxidant enzymes, GPx is considered to serve as the first antioxidant defense line [13]. Selenium (Se) is an essential trace element that functions as a cofactor of GPx; thus, it may play a vital role in the liver’s antioxidant defense system. Previous studies have demonstrated that Se supplementation may alleviate liver injury and inhibit the progression of preneoplastic lesions to large foci by reducing angiogenesis and apotosis-related signaling pathways, and lipid peroxide levels while simultaneously increasing antioxidant enzyme activities, such as those of superoxide dismutase and catalase [14,15]. However, the mechanism underlying the attenuation of HCC development by Se’s connection to GPx remains controversial [16,17].

In addition to the individual roles that GSH and Se play in HCC development, their potential synergistic effects on HCC development cannot be ignored. Therefore, this study aimed to explore the impact of GSH and Se supplementation, individually or in combination, on oxidative stress and GSH-related antioxidant capacities in DEN-induced hepatocarcinogenic mice.

## 2. Results

### 2.1. Body Weight and Liver Function Serum Markers

Table 1 presents the mice’s initial and final body weights as well as their liver and renal function markers across the five groups. The mice exhibited similar initial body weights among the five groups. After 20 weeks of treatment, DEN mice displayed a notable reduction in body weight compared with control mice, while DEN mice supplemented with GSH or Se exhibited an attenuated decrease in body weight. The percentage of liver weight in relation to body weight increased in all DEN-treated groups (DEN, DEN + GSH, DEN + Se, and DEN + GSH + Se) compared with that in the control group. Furthermore, liver function markers, such as serum aspartate aminotransferase (AST), alanine aminotransferase (ALT), and γ-glutamyl transferase (GGT) levels, were significantly higher in the DEN group than in the control and DEN supplemented with the GSH or Se groups. All the mice in the five groups yielded similar serum creatinine levels.

### 2.2. Pathological Classification of the Liver

**Figure 1:**
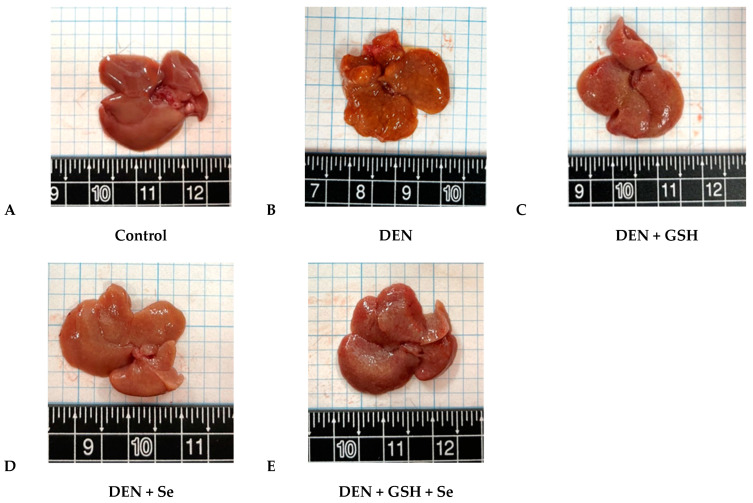
Gross findings of altered hepatocellular foci in mice at week 20. (**A**) Control group; (**B**) DEN group; (**C**) DEN + GSH group; (**D**) DEN + Se group; (**E**) DEN + GSH + Se group. DEN, diethylnitrosamine; GSH, glutathione; Se, selenium.

**Figure 2:**
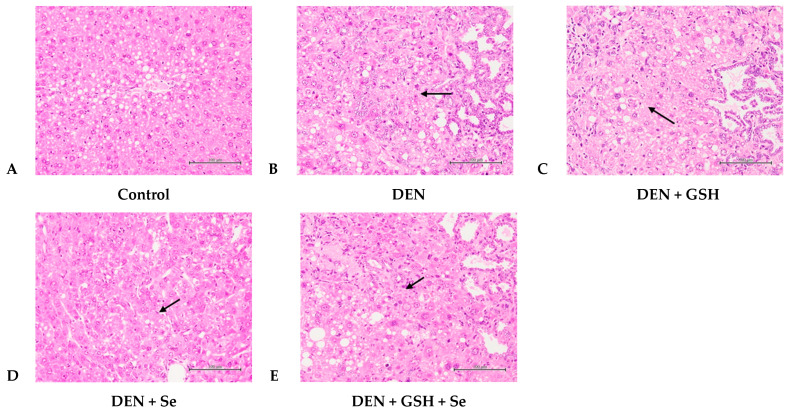
Histopathological changes of altered hepatocellular foci in mice at week 20 (400×). (**A**) Normal hepatocytes were observed in the control group. (**B**) Multifocal, moderately to severely altered hepatic foci (arrow), and moderate to severe bile duct and oval hyperplasia with inflammation and fibrosis were observed in the DEN group. (**C**–**E**) Multifocal, slightly to moderately altered hepatic foci (arrow), and slight to moderate bile duct and oval hyperplasia with inflammation and fibrosis. H&E stain, 400×. DEN, diethylnitrosamine; GSH, glutathione; Se, selenium.

Pathological grading of DEN mouse livers revealed moderate to severe degrees of hepatocellular alteration, proliferation of the bile duct, oval cell hyperplasia, and mild fibrosis, as illustrated in Figure 1B and Figure 2B. These histopathological changes corresponded to higher pathological scores for altered hepatic foci, hyperplasia, and fibrosis compared with those of the control group. In contrast, DEN + GSH, DEN + Se, and DEN + GSH + Se group mice displayed milder histopathological alterations and significantly lower pathological grades than DEN mice; however, these grades remained higher than those observed in the control group (Table 2). In particular, the DEN + GSH + Se group yielded slightly, but not significantly, lower pathological grades for altered hepatic foci, oval cell hyperplasia, and fibrosis than the DEN + GSH and DEN + Se groups (Table 2, Figure 1 and Figure 2).

### 2.3. Serum and Hepatic Oxidative Stress and Antioxidant Capacity Parameters

**Figure 3:**
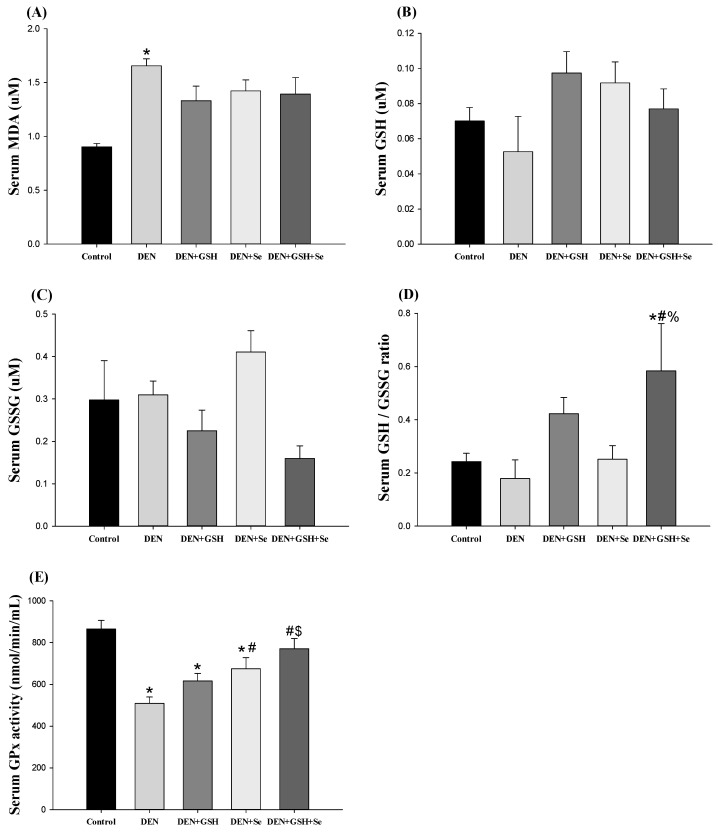
Serum oxidative stress and GSH-related antioxidant activity in mice with DEN-induced altered hepatocellular foci at week 20. (**A**) MDA, (**B**) GSH, (**C**) GSSG, (**D**) GSH/GSSG ratio, and (**E**) GPx activity were analyzed. Reported values are expressed as the mean ± SE (n = 6). * Indicates a significant difference from the control group; # indicates a significant difference from the DEN group; $ indicates a significant difference from the DEN + GSH group; % indicates a significant difference from the DEN + Se group (one-way ANOVA followed by the Student–Newman–Keuls test, *p* < 0.05). DEN, diethylnitrosamine; GSH, glutathione; Se, selenium; MDA, malondialdehyde; GSSG, glutathione disulfide; GPx, glutathione peroxidase.

The serum markers of oxidative stress and antioxidant capacity across the five groups are depicted in Figure 3. The DEN group exhibited significantly higher serum malondialdehyde (MDA) levels than the control group. Upon supplementation with GSH, Se, or both, these levels decreased and became similar between the DEN-treated and control groups (Figure 3A). Although serum GSH and GSSG levels remained consistent across all groups (Figure 3B–C), the DEN + GSH + Se group had a significantly higher serum GSH/GSSG ratio than the control, DEN, and DEN + Se groups and a similar ratio to the DEN + GSH group (Figure 3D). Combined supplementation with GSH and Se not only attenuated the DEN-induced decrease in serum GPx activity compared with the DEN and DEN + GSH treatments but also yielded similar GPx activity to that observed in control mice (Figure 3E).

**Figure 4:**
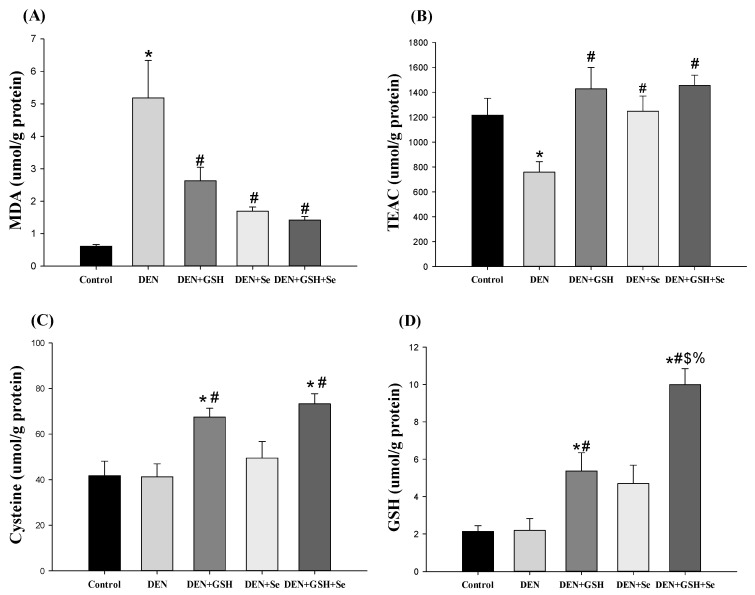

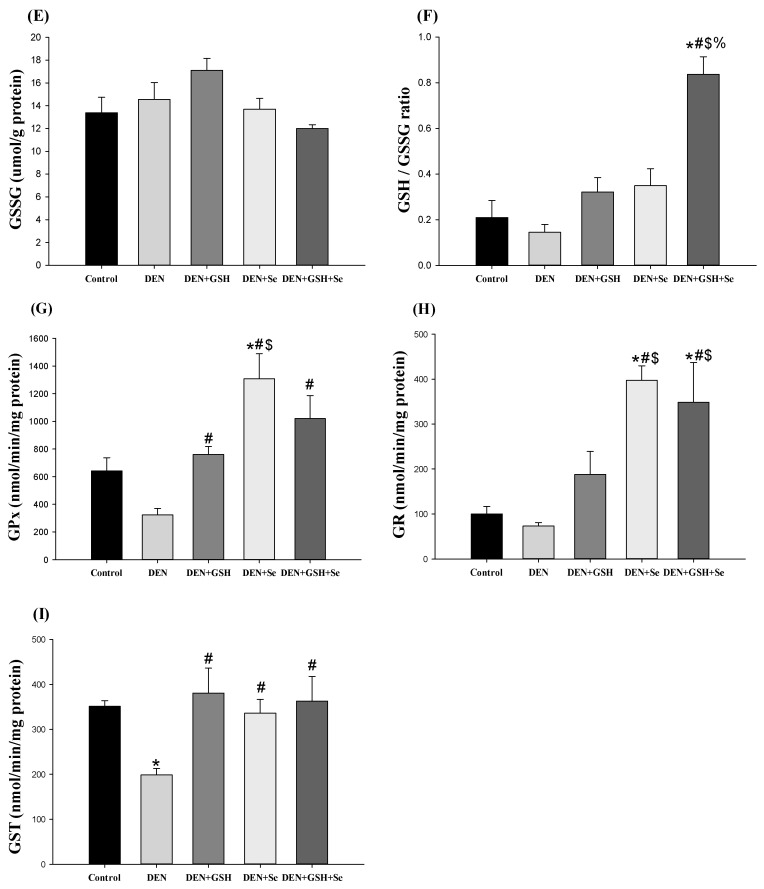
Oxidative stress and GSH-related antioxidant activity in the liver tissue of mice with DEN-induced altered hepatocellular foci at week 20. (**A**) MDA, (**B**) TEAC, (**C**) Cysteine, (**D**) GSH, (**E**) GSSG, (**F**) GSH/GSSG ratio, (**G**) GPx activity, (**H**) GR activity, and (**I**) GST activity were analyzed. Reported values are expressed as the mean ± SE (n = 6). * Indicates a significant difference from the control group; # indicates a significant difference from the DEN group; $ indicates a significant difference from the DEN + GSH group; % indicates a significant difference from the DEN + Se group (one-way ANOVA followed by the Student–Newman–Keuls test, *p* < 0.05). DEN, diethylnitrosamine; GSH, glutathione; Se, selenium; MDA, malondialdehyde; TEAC, trolox equivalent antioxidant capacity; GSSG, glutathione disulfide; GPx, glutathione peroxidase; GR, glutathione reductase; GST, glutathione S-transferase.

As demonstrated in Figure 4, oxidative stress, GSH, and its related antioxidant enzyme activity in liver tissue were compared among the five treatment groups. DEN mice supplemented with GSH, Se, or both exhibited significantly lower MDA levels but higher trolox equivalent antioxidant capacity (TEAC) levels in liver tissue compared with DEN mice; nonetheless, these levels were similar to those of control mice (Figure 4A,B). GSH supplementation in DEN mice significantly increased liver cysteine and GSH levels compared with other treatments (Figure 4C,D). However, a higher GSH/GSSG ratio was observed in the DEN + GSH + Se group (Figure 4F). Hepatic GPx, GR, and GST activities were slightly or significantly lower in DEN mice than in control mice (Figure 4G–I). Supplementation with GSH and Se in DEN mice elicited activities comparable to or greater than those observed in control mice.

## 3. Discussion

Consistent with previous studies [7,14], we successfully induced moderate to severe alteration of hepatic foci accompanied by elevated serum markers of liver function, as well as severe disequilibrium of oxidative stress and antioxidant capacities after chronic DEN administration in the present study. DEN is a chemical carcinogen widely used to induce hepatocarcinogenesis in rodent models. DEN administration to animals results in liver fibrosis, necrosis, altered hepatic foci, and eventually HCC formation [18,19]. DEN is primarily metabolized and detoxified in the liver, where it is converted to alkylating metabolites by cytochrome P450 enzymes that further induce DNA damage as well as stimulate excessive oxidative stress and chronic inflammation, mimicking the mechanism of human HCC development [18,19]. Although DEN-induced damage is mainly in the liver, the possibility of renal damage with high doses of DEN is also a concern [20]. Therefore, we examined serum creatinine levels as a marker of renal function and did not observe a difference between groups, indicating that 75 mg/kg DEN mainly induced hepatocarcinogenesis but did not cause detectable renal damage. Fortunately, supplemented with GSH and Se, especially combined supplementation, could reverse these phenomena and alleviate DEN-induced hepatocarcinogenesis.

Basically, orally administered GSH cannot be directly absorbed. It must be digested into glutamine, cysteine, and glycine, which are subsequently absorbed by the intestinal tract, transported to the liver, and used to resynthesize GSH in the liver; the GSH is subsequently exported to the systemic system [21,22]. Therefore, the bioavailability, transportation, and biosynthetic capacity of GSH in the liver potentially affects the efficacy of its supplementation. Kovacs-Nolan et al. [23] reported treating 10 mg of GSH to 16 ~ 20 g mice, equivalent to approximately 500 mg/kg, and observed that oral GSH supplementation could accumulate in the liver but was minimally reflected in plasma. Consistent with the previous study [23], we found non-significant trends of higher serum levels of GSH and GSH/GSSG ratio, while significantly higher hepatic levels of cysteine, GSH, GSH/GSSG ratio, and GSH-related antioxidant enzyme activities in GSH-supplemented groups. Richie et al. [24] reported that the efficacy of oral GSH supplementation on erythrocyte and plasma GSH levels depends on dose and duration, as they found the highest plasma GSH level in the high-dose group (1000 mg/d) after 6-month GSH supplementation; nevertheless, the levels returned to baseline after a 1-month period of cession in 54 non-smoking adults. A small-scale clinical study that administered oral GSH supplementation (50 mg/kg) to healthy human volunteers (equal to 3000 mg/60 kg human) did not observe an increase in serum GSH level [25]. Although we supplemented our study mice with a large dose of GSH (500 mg/kg), which was equivalent to that of 2500 mg/d for a 60 kg human [26], it had a hepatoprotective role in mice; the protective efficacy of GSH supplementation with this dose applied to humans needs further exploration. 

When the liver is damaged (e.g., infection with the hepatitis B and C virus, chemical exposure, etc.), chronic inflammation and excessive oxidative stress occur in liver cells [27]. GSH supplementation to increase GSH-related antioxidant activity may decrease lipid peroxidation and suppress inflammation and apoptosis to prevent hepatotoxicity and further fibrotic and carcinogenetic changes [28]. Studies have demonstrated that GSH supplementation may increase serum and liver GSH levels but decrease serum DNA peroxidation product and ALT levels, further assuaging liver damage in patients with nonalcoholic fatty liver disease [29,30]. However, the beneficial effect of increasing GSH levels on liver tumor suppression has exclusively been observed in the tumor initiation stage but not in the tumor progression stage, indicating that an earlier reductive environment in the liver can prevent HCC initiation upon carcinogen stimulation [8]. Rather, when liver tumorigenesis is in progress, GSH supplementation reduces intracellular ROS levels and desensitizes the Nrf2/GCLC antioxidant signaling pathway, thus increasing the incidence of HCC [9]. Qi et al. [8] also demonstrated that Sirtuin-1 liver-specific knockout mice showed resistance to DEN-induced HCC development through increasing the expression of Nrf2, subsequently elevating the GSH level to produce a reductive environment. Therefore, while high-dose oral GSH supplementation potentially increases GSH and its related antioxidant enzyme activity, we need to carefully monitor the possible harmful role of GSH in HCC progression. Inspiringly, in our study on mice, GSH supplementation initiated 1 week before DEN administration and continued for 20 weeks potentially exerted a protective rather than harmful effect on hepatocarcinogenesis. Echoing our finding, a recent study reported that HepG2 cells pretreated with GSH under physiological conditions potentially upregulated liver function, such as alcohol metabolic processes as well as cell proliferation, growth, and differentiation signaling pathways that regulate various cellular processes [31]. Furthermore, when GSH-pretreated HepG2 cells encounter oxidative stress, GSH potentially plays a hepatoprotective role by activating the signaling pathway related to Nrf2 and hypoxia-1 inducer factor 1, and the expression of GPx, GR, GST, and VEGF, while downregulating the expression of ALT and AST and disease-related pathways, including chemically induced ROS and fibrosis [31]. Therefore, we hypothesized that GSH may play a protective role during the progression of liver damage.

In addition to that of GSH, the protective effect of organic Se supplementation has proven to attenuate the development of HCC [14,15,32]. However, excessive supplementation with inorganic Se, such as sodium selenite, does not reduce but rather promotes tumor formation [33], probably because of selenite’s metabolic process, which aggravates oxidative stress. In this study, Se supplementation in organic form, seleno-L-methionine, moderated the pathological severity of HCC by increasing GPx and GR activities and reducing MDA levels. Our results are consistent with the observations made by Rohr-Udilova et al. [17], who found that decreasing Se levels, causing excessive lipid peroxides, would activate the transcription factor activator protein 1, which regulates angiogenesis and inflammation pathway, to increase vascular endothelial growth factor and interleukin -8, thus promoting HCC tumor progression in vitro, in vivo, and clinical research. Se supplementation could inhibit HCC through upregulating GPx4 levels to modulate lipid peroxides levels [17]. Wu et al. [34] reviewed numerous in vitro and in vivo studies and summarized that Se supplementation, especially in organic form, serves a preventive role in altered hepatic foci and hepatic tumor formation by balancing oxidative stress and antioxidant activity as well as modulating inflammation, proliferation, angiogenesis, and apoptosis related signaling pathways. The role of Se in HCC development cannot be ignored.

In this study, we implemented combined supplementation with GSH and Se in mice, exhibiting a similar protective effect compared to that with individual GSH or Se supplementation, although the lowest pathological gradings for altered hepatocellular foci, hyperplasia, and fibrosis change as well as serum levels of AST and GGT were observed. This combined effect possibly emanates from the simultaneous increase in GSH and its related enzymes, including GPx, GR, and GST, thus enhancing antioxidant activity. Experimental research found that supplementation with Se–GSH-enriched probiotics exerted a better protective effect on carbon tetrachloride-induced liver fibrosis than that with Se, GSH, or probiotics individually, as it increased GSH-related antioxidant activity and upregulated the silent information regulator 1 signaling pathway, thus helping reduce hepatic oxidative stress and inflammation [35]. A previous study demonstrated that co-supplementation with GSH and selenite increased GPx activity and inhibited chemically induced skin tumor promotion [36]. To our knowledge, this is the first study to explore the impact of GSH and Se co-administration on alleviating hepatocarcinogenesis, and its positive results yield a potential target for HCC prevention in future studies.

However, this study has certain limitations. First, although we successfully detected pathological changes in altered hepatic foci and oval cell hyperplasia, we did not identify HCC after DEN administration, probably because of the short duration of our experiment. Previous studies have typically administered a DEN injection once in 15-day-old mice and observed their incidence of HCC at 35 weeks [37]. We employed a two-step induction process entailing a 6-week cancer-initiating period and a 6-week cancer-promoting period to induce the development and progression of hepatocarcinogenesis. Therefore, we speculated that the time interval between the cancer-promoting period and the end of our experiment might have been too short to observe the formation of HCC. Future studies should employ a longer observation period after promoting tumor formation. Second, evaluating protein expression in the liver architecture using immunohistochemistry (IHC) method was adopted to observe the evidence of HCC in previous research. For instance, glutathione *S*-transferase placental-1 (Gstp1) is a commonly used marker to evaluate HCC in mice [38], while cytokeratin-19 (CK19) is a marker of early mouse neoplastic lesions [39]. Unfortunately, we did not examine the expression Gstp1 and CK19 in the liver owing to our limited sample size. Third, we did not explore the signaling pathway underlying the protective effect of GSH and Se on hepatocarcinogenesis owing to our limited sample size. However, the Nrf2–Kelch-like ECH-associated protein 1 signaling pathway was possibly involved in the present study, as it has been shown to be the main regulator of GSH-related antioxidant activity in a previous study [40]. Further exploration is required to confirm this assumption.

## 4. Materials and Methods

### 4.1. Reagents and Diets

DEN (Cat. N0258-1G), GSH (Cat. G4251-25G), and seleno-L-methionine (Cat. G4251-100MG) were purchased from Sigma-Aldrich (St. Louis, MO, USA). Commercial assay kits for GSH and GSSG level, and GST activity analyses were obtained from Abcam (Cambridge, UK), and a GPx activity assay kit was acquired from Cayman Chemical Company (Ann Arbor, MI, USA).

To mitigate high-dose Se interference in the diet, the 58M1 TestDiet^®^ (Richmond, IN, USA) was procured. This diet is a modification of the AIN-93M semi-purified diet, comprising 73% carbohydrate, 13% protein, and 4% fat, with sufficient essential vitamins and minerals, except Se, which was eliminated at 0.1 ppm, a figure approximately comparable to the Recommended Dietary Allowance for adult humans as reported by Kasaikina et al. [33].

### 4.2. Animal Study

Three-week-old C57BL/6J mice were secured from the National Laboratory Animal Center (Taipei, Taiwan) and housed in the animal room of Chung Shan Medical University (Taichung, Taiwan) under the following controlled conditions: temperature (22–24 °C), humidity (55–60%), 12:12 light/dark cycle, and ad libitum access to food and water. After 1-week acclimatization, the mice were randomly assigned to the following five groups for a duration of 20 weeks: control, DEN, DEN + GSH, DEN + Se, and DEN + GSH + Se (n = 6 per group). GSH (500 mg/kg) and Se (350 μg/kg) were administered via oral gavage daily from week 1 until the end of the study, as described previously [23,33]. The DEN administration protocol was adopted from the study of Kushida et al. [39] and Memon et al. [41]. DEN (75 mg/kg dissolved in a 0.9% saline solution) was intraperitoneally administered once weekly from week 2 to week 9 of the experimental period as a cancer-initiating agent. After a 6-week discontinuation period, DEN was re-administered once weekly from week 16 to week 19 of the experimental period as a cancer-promoting agent. Mice were fasted overnight and subsequently euthanized via CO_2_ asphyxiation at week 20 of the treatment period.

**Figure 5:**
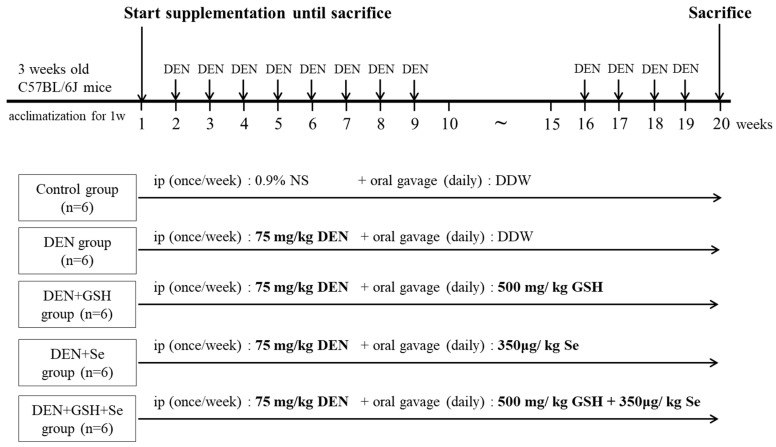
Experimental protocol.

Blood samples were collected using anticoagulant-free vacutainers (Becton Dickinson, Rutherford, NJ, USA) and subsequently centrifuged at 806× *g* for 10 min at 4 °C to obtain serum samples. Liver tissue was isolated and weighed, and its appearance was recorded via immediate photographing. A part of the liver sample was subjected to histopathological examination and the remainder homogenized in 10 times the weight of radioimmunoprecipitation assay buffer (150 mM sodium chloride, 50 mM Tris-HCl, 1% Nonidet^®^ P-40, 0.5% sodium deoxycholate, and 0.1% sodium dodecyl sulfate) on ice. The homogenates were centrifuged at 8870× *g* for 10 min at 4 °C and the supernatants stored in freezers at −80 °C until analysis. Daily health checks were performed, and the body weights and food intakes of the mice were recorded three times a week throughout the experiment. All researchers in this study underwent the Animal Care and Use Training Program provided by the Animal Care and Use Center of Chung Shan Medical University. The detailed study protocol is depicted in Figure 5.

### 4.3. Pathological Evaluation

The largest right lobe of the liver tissue from each mouse was isolated, fixed in 10% formalin overnight, and embedded in paraffin. The paraffin-embedded specimens were sliced to 5-µm sections and stained with hematoxylin and eosin dye for histopathological examination. Each liver tissue sample was examined under a light microscope. The grading of altered hepatic foci, hyperplasia, and fibrosis was evaluated and classified based on tissue severity using the methods described by Shackelford et al. [42]. Severity was graded on a scale of 1 to 5 degrees, representing minimal to severe changes: 1, minimal (<1%); 2, slight (1–25%); 3, moderate (26–50%); 4, moderate/severe (51–75%); and 5, severe/high (76–100%). Pathological evaluations were conducted by a qualified pathologist from the Graduate Institute of Veterinary Pathobiology, National Chung Hsing University, Taichung, Taiwan.

### 4.4. Biochemical Measurements

Serum samples were utilized to measure the levels of ALT, AST, GGT, creatinine; MDA, as an oxidative stress inducer; antioxidant indicators, including TEAC, GSH, GSSG, and the GSH/GSSG ratio, and GPx activity. Liver tissue homogenates were subjected to MDA, TEAC, cysteine, GSH, GSSG, and GSH/GSSG ratio evaluations as well as GPx, GR, and GST activity analyses.

MDA levels were measured using the thiobarbituric acid method described by Lapenna et al. [43]. Commercial assay kits were used to analyze GSH and GSSG levels as well as GST and GPx activities according to the manufacturer’s instructions. GR activity was measured using the method described by Carlberg et al. [44]. Briefly, we measured β-nicotinamide dinucleotide phosphate (NADPH) oxidation to NADP^+^ at a 340 nm wavelength for 1 min to determine GR activity, since GR functions to catalyze NADPH oxidation to NADP^+^, while simultaneously reducing GSSG to GSH. Cysteine levels were analyzed using high-pressure liquid chromatography (HPLC) with 0.1 M potassium dihydrogen phosphate solvent containing 2% acetonitrile and a LiChrospher^®^ 100 RP-18 column obtained from Merck (Darmstadt, Germany). TEAC levels were evaluated by measuring the reduction of free radicals (ABTS+) to ABTS at a 374 nm wavelength using trolox as a standard [45]. Tissue protein concentrations were determined using the Bradford assay, with bovine serum albumin as the standard.

### 4.5. Statistical Analysis

Data were analyzed and plotted using SigmaPlot software (version 12.5; Systat Software Inc., Chicago, IL, USA). One-way analysis of variance, followed by the Student–Newman–Keuls test, was used for comparisons among groups. Data are presented as the mean ± standard error of the mean (SE), with statistical significance set at *p* < 0.05.

## 5. Conclusions

DEN administration causes a pathological change in hepatocarcinogenesis by increasing oxidative stress and decreasing GSH-related antioxidant activity. Supplementation with GSH, Se, or both potentially attenuates DEN-induced hepatocarcinogenesis by restoring GSH and its related antioxidant enzymes (i.e., GPx, GR, and GST), thus counterbalancing excessive oxidative stress. 

## Figures and Tables

**Table 1:** Effects of GSH and Se on the body weights and serum hepatic and renal function markers of mice with DEN-induced altered hepatocellular foci on week 20 ^1^.

	Control	DEN	DEN + GSH	DEN + Se	DEN + GSH + Se
Initial BW(g)	10.2 ± 0.5	11.7 ± 0.5	11.5 ± 0.2	11.3 ± 0.2	11.8 ± 0.4
Final BW(g)	26.4 ± 0.7	20.0 ± 0.4 *	22.1 ± 0.3 *^,#^	22.1 ± 0.5 *^,#^	21.3 ± 0.5 *
Liver weight (g)	0.9 ± 0.0	1.0 ± 0.0	1.0 ± 0.0	1.0 ± 0.0	0.9 ± 0.1
Liver (% BW)	3.28%	4.79% *	4.49% *	4.32% *	4.24% *
AST (U/L)	153.5 ± 7.2	282.7 ± 14.1 *	269.5 ± 19.3 *	213.3 ± 15.3 *^,#^	205.3 ± 18.5 *^,#^
ALT (U/L)	15.3 ± 1.1	77.0 ± 2.1 *	69.7 ± 2.2 *	55.0 ± 3.7 *^,#^	57.7 ± 3.4 *^,#^
GGT (U/L)	22.3 ± 1.0	31.7 ± 3.4 *	24.7 ± 1.5 ^#^	22.3 ± 0.8 ^#^	19.7 ± 1.0 ^#^
Creatinine (mg/dL)	0.2 ± 0.0	0.2 ± 0.0	0.2 ± 0.0	0.2 ± 0.0	0.2 ± 0.0

^1^ n = 6 per group. Values are expressed as the mean ± standard error of the mean. * Indicates a significant difference from the control group; ^#^ indicates a significant difference from the DEN group (one-way ANOVA followed by the Student–Newman–Keuls test, *p* < 0.05). DEN, diethylnitrosamine; GSH, glutathione; Se, selenium.

**Table 2:** Pathological grading of altered hepatocellular foci in DEN-treated mice at week 20 ^1^.

Histopathological Findings ^2^	Control	DEN	DEN + GSH	DEN + Se	DEN + GSH + Se
Altered hepatic foci	0 ± 0	4.3 ± 0.2 *	2.7 ± 0.2 *^,#^	2.8 ± 0.2 *^,#^	2.5 ± 0.2 *^,#^
Eosinophilic type	0 ± 0	1.0 ± 0.0 *	1.0 ± 0.0 *	1.0 ± 0.0 *	1.0 ± 0.0 *
Basophilic type	0 ± 0	1.0 ± 0.0 *	0.8 ± 0.2 *	1.0 ± 0.0 *	0.5 ± 0.2 *
Clear type	0 ± 0	3.3 ± 0.2 *	2.3 ± 0.3 *^,#^	2.8 ± 0.2 *	2.5 ± 0.2 *^,#^
Bile duct hyperplasia	0 ± 0	2.5 ± 0.2 *	2.0 ± 0.3 *	2.0 ± 0.0 *	1.7 ± 0.2 *^,#^
Oval cell hyperplasia	0 ± 0	3.7 ± 0.2 *	2.8 ± 0.2 *^,#^	3.0 ± 0.3 *^,#^	2.7 ± 0.2 *^,#^
Fibrosis	0 ± 0	1.7 ± 0.2 *	1.7 ± 0.2 *	1.3 ± 0.2 *	1.3 ± 0.2 *

^1^ n = 6 per group. ^2^ Degree of the lesion was graded from one to five depending on severity: 1, minimal (<1%); 2, slight (1–25%); 3, moderate (26–50%); 4, moderate/severe (51–75%); 5, severe/high (76–100%). The final numerical score was calculated by dividing the sum of the number of affected mice per grade by the total number of examined mice. Values are expressed as the mean ± standard error of the mean. * Indicates a significant difference from the control group; ^#^ indicates a significant difference from the DEN group (one-way ANOVA followed by the Student–Newman–Keuls test, *p* < 0.05). DEN, diethylnitrosamine; GSH, glutathione; Se, selenium.

## Data Availability

Data described in the manuscript will be made available upon request pending application and approval.

## Abbreviations

ALT: alanine aminotransferase; AST, aspartate aminotransferase; DEN, diethylnitrosamine; GGT, γ-glutamyl transferase; GPx, glutathione peroxidase; GR, glutathione reductase; GSH, glutathione; GSSG, glutathione disulfide; GST, glutathione S-transferase; HCC, hepatocellular carcinoma; MDA, malondialdehyde; NAC, N-acetylcysteine; NADPH, β-nicotinamide dinucleotide phosphate; ROS, reactive oxygen species; Se, selenium; TEAC, trolox equivalent antioxidant capacity.
